# Mindful monitoring and accepting the body in physical activity mediates the associations between physical activity and positive body image in a sample of young physically active adults

**DOI:** 10.3389/fspor.2024.1360145

**Published:** 2024-04-05

**Authors:** Rasa Jankauskiene, Migle Baceviciene

**Affiliations:** ^1^ Institute of Sport Science and Innovations, Lithuanian Sports University, Kaunas, Lithuania; ^2^ Department of Physical and Social Education, Lithuanian Sports University, Kaunas, Lithuania

**Keywords:** state mindfulness, physical activity, Monitor and Acceptance Theory (MAT), positive body image, embodiment

## Abstract

**Introduction:**

The study aimed to extend research on the possible mechanisms that explain the associations between physical activity (PA), mindfulness during PA and positive body image. In the present study, we tested the mediating role of state mindfulness during PA in the association between PA and trait body appreciation. We also explored the moderating roles of sex and body mass index (BMI) in these associations.

**Methods:**

In total, 759 students participated in the study but after applying the inclusion criteria 539 questionnaires were approved for analysis, with a mean age of 23.3 ± 7.2 years (49.7% were women). Study participants completed the online survey, which included sociodemographic details, PA data, the State Mindfulness Scale for Physical Activity 2 (SMS-PA-2) and the Body Appreciation Scale 2 (BAS-2).

**Results:**

Monitoring and accepting the body during exercise mediated the association between PA and body appreciation. BMI moderated the association between accepting the body during exercise and body appreciation: for heavier individuals with BMI > 24.99 kg/m^2^, the associations between accepting the body during exercise and body appreciation were higher (*B* = 0.53, *p* < 0.001) compared to those whose body weight was in the normal range (*B* = 0.21, *p* < 0.001).

**Conclusion:**

Strengthening mindfulness and promoting mindful body acceptance during exercise might enhance a positive body image, especially in individuals with a higher BMI.

## 1 Introduction

Mindfulness is described as self-regulation of attention, awareness, monitoring and accepting the mind and body and the reduction of cognitive and emotional reactivity (1, 2). Mindfulness might be conceptualized as both a reliable individual difference (dispositional or trait mindfulness) and as a momentary experience that can vary within individuals across time and context (state mindfulness) (3). Weak to moderate correlations were observed in associations between trait mindfulness and more frequent PA (4). However, there is less research on testing the relationships between PA and state mindfulness.

The mechanisms through which trait mindfulness is related to PA are based on improved self-control through increased attention and acceptance, improved self-regulation, increased satisfaction and more autonomous motivation (5–10). Attention and acceptance, as cognitive characteristics, might have the ability to override or inhibit unhealthy habits. Specifically, mindful awareness of PA could enhance the acceptance of negative and uncomfortable thoughts and sensations that might occur during PA, especially in novice exercisers, individuals with body image concerns and the overweight (i.e., shame, discomfort, pain, fatigue, exertion). Increased self-control, improved attention and awareness leading to higher self-regulation, greater satisfaction and more autonomous exercise motivation might help to encourage people to sustain PA in both the short and long term.

Systematic studies revealed that positive body image is related to higher PA and greater body functionality appreciation (11–13). Possible pathways that explain increased positive body image as an outcome of participation in PA include increased objective and subjective physical fitness, self-efficacy (increased empowerment and feeling of competence and control), lower body surveillance and self-objectification and an increase in body functionality appreciation (14–17). Research show that PA positively affects body image only in the case of autonomous motivation (18). Mechanisms through which PA positively affects body image are not yet fully understood since most of the studies are atheoretical (11).

Some PAs are considered to be more mindful compared to other activities. For example, yoga was considered mindfulness-based PA because it focuses on mindful-based movements, mindful breathing, focused attention to the body, contains mindful components by being process-oriented and emphasizes body and mind connection (19). Results of previous studies suggested that mindfulness-based PAs such as yoga might have an enhancing effect on positive body image and physical self-perception (15, 17, 20–23). Important prospective study demonstrated that yoga participation decreased self-objectification (treating self as an object to be viewed and evaluated based on appearance) and increased self-concept, internal reasons for exercise (health and fitness) and state mindfulness during PA (20).

However, despite the evidence that some PAs are more mindful, it is reasonable to assume that all PA might be associated with higher general mindfulness compared to a sedentary lifestyle. Physical inactivity and a sedentary lifestyle might be seen as practices that neglect and ignore the authentic needs of the body. PA and sports are contexts in which the body has a central focus of an individual's lived experience and it is an ideal way to experience it (14). A professionally prescribed and delivered PA might increase concentration, strength, stamina and coordination and give rise to an internally oriented experience of the body through more frequent states of mind–body integration and positive embodiment (16).

Based on the Developmental Theory of Embodiment (24) and/or the Embodiment Model of Positive Body Image (14, 16), embodiment is an internally oriented state of mind–body connection in which one experiences one's body as an essential aspect of interrelated experiences of competence, relatedness to others, power, self-expression, vitality and well-being. It places a person more in tune with the body's sensations and more appreciative of body functions (24). Positive embodiment is in contrast to the self-objectification, body surveillance and societal focus on appearance that is associated with dysfunctional eating and exercise (25). In highly body image-concerned individuals, health-damaging PA patterns beyond the normal frequency and intensity are observed, including exercising for permission to eat, exercising in the absence of proper nutrition/hydration and exercising when injured and feeling pain, exercising for self-punishment or harm (26). In other words, in dysfunctional exercise individuals demonstrate low levels of body awareness, ignore their feelings and thoughts or neglect body needs.

In mindfulness, the internal aspects of the self (i.e., feelings, thoughts, physiological needs) are well represented (1). In mindful PA, physical movements are implemented with attention, purpose, self-compassion, acceptance, awareness and joy. Mindful PA is focused on the process of becoming more connected, healthier and stronger, whereas mindless exercise is often appearance-based and focused on outcomes (26–28). In PA, people might demonstrate different levels of monitoring, awareness and acceptance of their feelings, thoughts and physical sensations of the body, thus possessing different levels of mindfulness (4, 29). Mindful awareness and acceptance in PA means that individuals are aware and accept the inner aspects of the self (body, thoughts and feelings) in a non-judgmental way.

Recently, the Monitor and Acceptance Theory (MAT) was developed (30). This theory postulates that mindfulness includes two components of attention monitoring and acceptance, which together might explain its impact on psychological well-being. Attention monitoring skills enhance the awareness of present-moment internal and external experiences and affect positive cognitive outcomes, affective experience and reactivity. Acceptance skills modify the way one relates to the present-moment experience, regulating reactivity to affective experience (30, 31). MAT is an important theory that might help to explain the role of mindfulness in PA and its correlates (32). Open and non-judgmental awareness (monitoring) of physical body sensations during PA might support feelings of confidence in the body, as well as general feelings of competence and autonomy.

Novice exercisers, overweight individuals and persons with body image concerns might feel discomfort in PA and thus body and mind monitoring skills might help them to regulate the intensity of PA or the content of exercises fostering feelings of autonomy, competence and self-control. Acceptance of negative or uncomfortable thoughts or body sensations (i.e., body shame, fatigue, pain, exertion, etc.) during PA might help novice exercisers and individuals with body image concerns to sustain PA both in the short and long term (4). Mindful PA and positive body image might be connected in reciprocal and self-perpetuating ways. For example, mindful PA might increase positive feelings about one's own body and these positive feelings might increase PA. Thus, it is important to understand the role of mindfulness in the associations between PA in general and positive body image. This knowledge might inform PA, positive body image promotion-focused interventions as well as prevention and treatment programmes for individuals which body weight is higher than normal range.

There are no data reporting significant sex differences in mindfulness during PA. However, men and people with body mass index (BMI) of normal ranges report higher positive body image and less body dissatisfaction compared to women and those which body weight is higher than normal ranges (33–35). It is explained by different sociocultural expectations for sexes and stronger pressures to attain appearance ideals for women compared to men and for people with different than stereotyped body images (36). Also, PA in men is higher compared to women, especially in young adult age (37). Therefore, sex and BMI might moderate the associations between PA, mindfulness, and positive body image. No previous studies tested moderating role of sex and BMI in the associations between PA, mindfulness during PA and positive body image.

**Figure 1:**
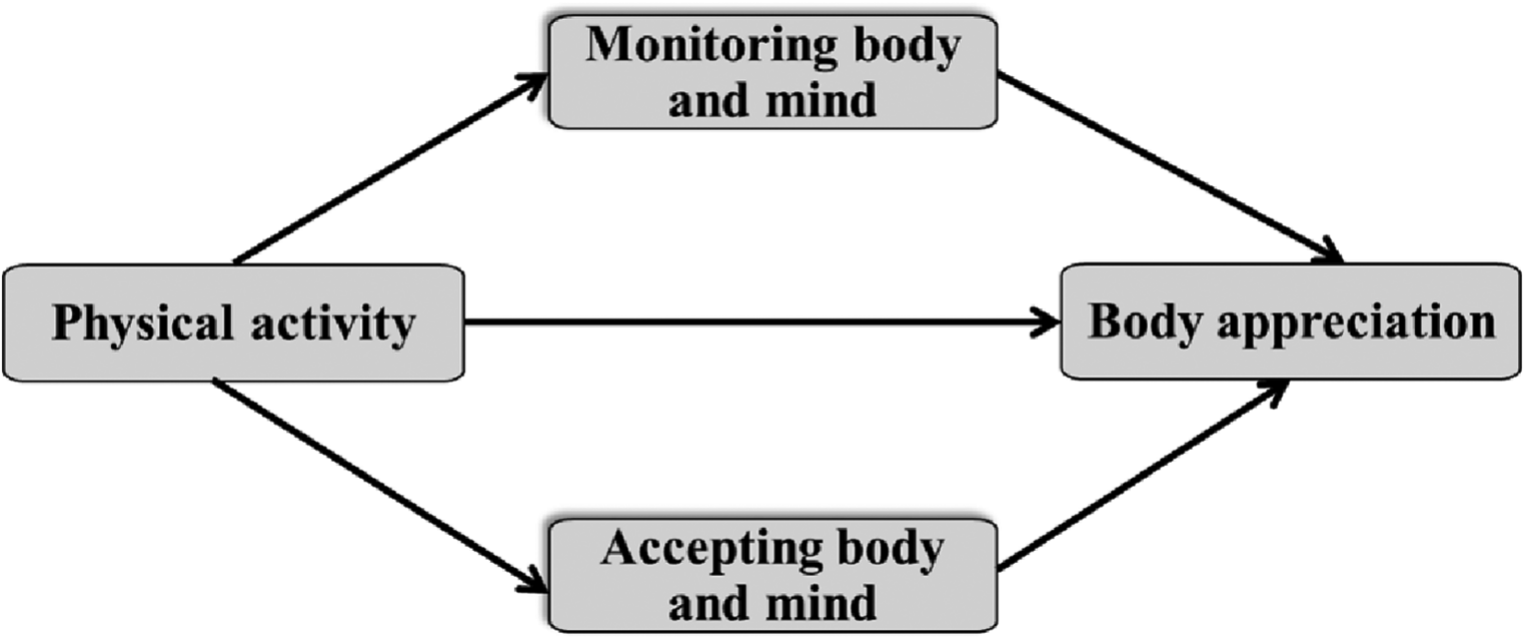
Hypothetical model of the study.

Based on previous studies, the present study aims to extend research on the possible mechanisms that explain the associations between PA, mindfulness and positive body image. Specifically, the aim of the present study was to test the mediating role of mindful monitoring and acceptance of mind and body during PA in the associations between PA and positive body image (operating as body appreciation, Figure 1). In the present study, we expect that mindfulness (monitoring and accepting body and mind) during PA will mediate the associations between PA and positive body image. The second aim was to test the moderating role of sex and BMI in the associations between the variables of the model. We hypothesized that the associations between PA, mindfulness and positive body image might be stronger in women compared to men and in people with the higher BMI compared to those which BMI is in normal ranges.

## 2 Materials and methods

### 2.1 Participants and procedures

The study was approved by the Social Research Ethics Board of the Lithuanian Sports University (Protocol No. SMTEK-131) and data were collected between November 2022 and May 2023. Study participants of the present study were technical, medical, health, social sciences and humanities students at Lithuanian universities and colleges. First, permission to implement the survey was obtained from tadministrative units of the universities and colleges. The non-probabilistic sampling method was used to recruit the students, with 759 meeting the inclusion criteria and thus participating in the survey. Only 539 questionnaires were confirmed for further analysis after applying the inclusion criteria (age ≥ 18 years, studying at university or college, Lithuanian language spoken, regular participation in any sport for no less than six months and participation in an exercise session no longer than two weeks ago). Students who confirmed not exercising, exercising for less than six months and not being able to recall their experience during the last exercise session were excluded from this study (*n* = 220). The mean age of the students was 23.3 ± 7.2 years (range: 18–44 years).

The online survey was implemented using the SurveyMonkey platform and a trained researcher distributed the link to the study participants. All questions were set as mandatory; thus, no missing data were observed. Only one response from the same IP address was accepted. After introducing the study aims, information about anonymity of the questionnaire and the approximate time needed for completing the questionnaire, students provided digital informed consent and were directed to the Measures section. The Measures section of the questionnaire included questions assessing positive body image, PA, state mindfulness in PA and BMI. Students who refused to participate or did not meet the inclusion criteria were eliminated from the study. Students could end participation in the study at any point simply by closing their browser; in that case, their answers were not recorded.

### 2.2 Measures

The participants reported their sex, age, university, programme of studies and the nature of their PA (home exercise, recreational, organized individual sports, organized team sports). Furthermore, students were asked to report the time of their last exercise session.

State mindfulness during PA was assessed using the Lithuanian version of the State Mindfulness in Physical Activity Scale 2 (SMS-PA-2; 29). The 19-item instrument consists of four subscales: Monitoring Mind (6 items), Monitoring Body (6 items), Accepting Mind (3 items) and Accepting Body (4 items) in PA. Examples of items are: Monitoring Mind, “I noticed pleasant and unpleasant thoughts”; Monitoring Body, “I was aware of how my body felt”; Accepting Mind, “I let my thoughts/emotions just be without judging them”; and Accepting Body, “I accepted how my body felt even if it was unpleasant”. The scale uses a five-point Likert scale with responses ranging from “not at all” (1) to “very much” (5). The higher scores show the greater monitoring and/or accepting body and/or mind. The final score is calculated by averaging the response options for each subscale of the SMS-PA-2 and the total scale, thus it can vary in the range from 1 to 5. The Lithuanian version of the manuscript demonstrated acceptable psychometric properties (38). In the present sample, internal consistency was good, with Cronbach's alpha values of 0.87 for the general instrument, 0.83 for Monitoring Mind, 0.86 for Monitoring Body, 0.63 for Accepting Mind and 0.76 for Accepting Body.

PA was assessed using the Lithuanian version of the Leisure-Time Exercise Questionnaire (LTEQ; 39). The participants of the study reported the frequency of light, moderate and strenuous PA that lasted for no less than 15 min during the previous week. The frequency of light PA was multiplied by 3, moderate activity by 5 and strenuous activity by 9. The scores of each type of PA were summed to obtain the total PA score, with higher scores representing higher PA. The Lithuanian version of the instrument was applied previously for testing young adults (40).

Positive body image was assessed using the Lithuanian version of the Body Appreciation Scale 2 (BAS-2; 41). This scale is one-dimensional and consists of 10 items. Examples of the items are: “I respect my body” and “I appreciate the different and unique characteristics of my body”. The answers are scored on a Likert scale from 1 (*Never*) to 5 (*Always*) and then summed and averaged, with higher scores indicating higher body appreciation. The psychometric properties of the Lithuanian version of the questionnaire were good (42). Furthermore, the instrument demonstrated good scalar invariance across 65 nations (43). In the present study, Cronbach's alpha for the scale was good (*α* = 0.95).

The BMI was calculated using self-reported body weight (kg) and height (m): Weight/(Height)². In the student sample, the BMI ranged from 14.9 to 38.0 (mean = 23.1, SD = 3.3) kg/m². The criteria for classification of study participants into underweight (<18.5 kg/m^2^; 4.6%), normal weight (18.5–24.9 kg/m^2^; 71.8%), overweight (25.0–29.9 kg/m^2^; 20.4%) and obesity (≥30.0 kg/m^2^; 3.2%) categories were suggested by the World Health Organization (44).

### 2.3 Statistical analyses

Preliminary analyses, correlation analyses and testing of the variables' distribution normality and internal consistency of the scales were conducted using SPSS v.29 (IBM Corp., Armonk, NY, USA). A Cronbach's alpha value of >0.65 was considered to be adequate (45) but it should be noted that Cronbach's alpha values are sensitive to the number of items included in the scale (46). After confirming the distribution normality of all the continuous variables, Pearson's correlation coefficient was used to test the associations between study variables. Correlations of 0.1–0.3 were considered to be small, those of >0.3 and <0.5 as moderate and those of ≥0.5 as strong, with a significance level of <0.05 (47).

In addition, moderated mediation analysis was conducted using Mplus v.7.8 (Muthen & Muthen, Los Angeles, CA, USA). The calculated power for this sample (*n* = 539) was 0.98 for the model with Accepting Body and Monitoring Body as the two parallel mediators during exercise and 0.24–0.30 for the model with Accepting Mind and Monitoring Mind (48). PA was used as the independent variable (X), mindfulness in monitoring and accepting the body and mind during exercise as parallel mediators (M1, M2) and body appreciation as the dependent variable (Y). Finally, a moderating role of sex (male and female) and BMI groups (normal weight and overweight/obesity) was tested on all the paths of the mediation model: from X to M1 and M2, from M1 and M2 to Y and the directly from X to Y.

A bootstrapping procedure was used to test the significance of the total and indirect effects and the differences in these effects across levels of the moderator variables with 5,000 bootstrap samples (49). The 95% confidence intervals for the coefficients calculated by bootstrapping methods were considered statistically significant if the confidence intervals did not include 0.

## 3 Results

Five hundred thirty-nine (*n* = 539) individuals participated in study. From them 268 (49.7%) were women. Most of the sample comprised university students (90.2%) engaged in first-degree studies (86.8%) and having BMI in normal range (71.8%). All study participants were engaged in sports, with around half of them in recreational activities (50.5%). Other half of the sample (49.5%) participated in individual and/or organized team sports. Half of the study sample (50.2%) reported participation in sports on the same day as completing the survey or the day before, 26.3% two or three days before, 9.3% within one week, 14.2% within two weeks.

Pearson's correlation coefficients between study measures are presented in Table 1. A higher level of PA was positively and weakly correlated with the Monitoring Mind, Monitoring Body and Accepting Body subscales and body appreciation. Stronger associations were found between the body monitoring and acceptance and body appreciation (*r* = 0.43) than with the monitoring and accepting mind subscales (*r* = 0.16 and 0.21). Correlations between different SMS-PA-2 subscales demonstrated medium to strong correlations (0.29–0.56).

**Table 1:** Correlations between physical activity, state mindfulness in monitoring and accepting the mind and body and body appreciation (*n* = 539).

Study measures	BMI	PA	MM	AM	MB	AB	BA
Body mass index (BMI)	1.00						
Physical activity (PA)	−0.01	1.00					
Monitoring mind (MM)	−0.05	0.09*	1.00				
Accepting mind (AM)	0.05	0.06	0.32**	1.00			
Monitoring body (MB)	−0.03	0.18**	0.38**	0.29**	1.00		
Accepting body (AB)	−0.01	0.18**	0.32**	0.42**	0.56**	1.00	
Body appreciation (BA)	−0.12**	0.18**	0.16**	0.21**	0.43**	0.43**	1.00

* *p* < 0.05.

** *p* < 0.01; body appreciation was measured by the body appreciation scale 2; monitoring mind, accepting mind, monitoring body, and accepting body are subscales from the state mindfulness scale in physical activity 2.

**Figure 2:**
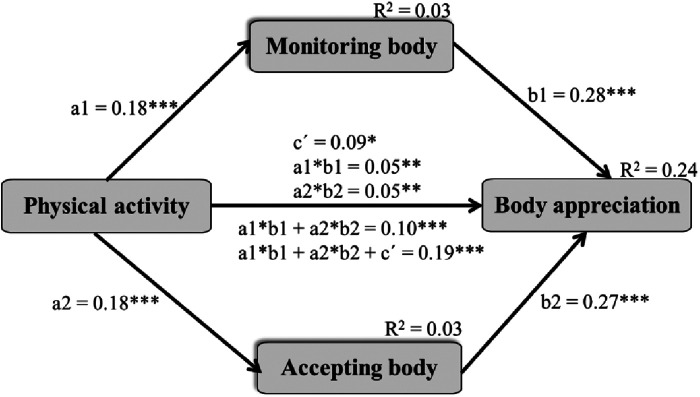
Mediated model of physical activity, mindfulness in monitoring and accepting the body during exercise and body appreciation. * *p* < 0.05, **p < 0.01, *** *p* < 0.001 two-tailed; coefficients are standardised; a1—effect from the independent variable (X) on mediator 1 (M1), b1—effect from M1 on the dependent variable (Y), a2—effect from X on M2, b2—effect from M2 on Y, ć—direct effect from X to Y, a1*b1—specific indirect effect of X on Y via M1 only, a2*b2—specific indirect effect of X on Y via M2 only, a1*b1 + a2*b2—total indirect effect of X on Y via M1, M2, a1*b1 + a2*b2 + ć—total effect.

In Figure 2, a mediated model of PA, mindfulness in monitoring and accepting the body during exercise and body appreciation is presented. Mindfulness in monitoring and accepting the body served as parallel mediators in the association between PA and body appreciation. Also, a direct association between PA and body appreciation was found. All information on direct, indirect and total effects is presented in Figure 2 footnotes. In addition, PA and monitoring and accepting the body during exercise explained 24% of the variance in body appreciation.

In the next step, we tested the moderated roles of sex and BMI on all the paths explored previously. There were no sex moderating roles on any path: from physical activity to monitoring the body during exercise (*β *= 0.17, *p* = 0.175), from physical activity to accepting the body (*β *= 0.25, *p* = 0.051), from monitoring the body to body appreciation (*β *= –0.31, *p* = 0.209), from accepting the body to body appreciation (*β *= 0.02, *p* = 0.942) and directly from physical activity to body appreciation (*β *= –0.13, *p* = 0.316).

**Figure 3:**
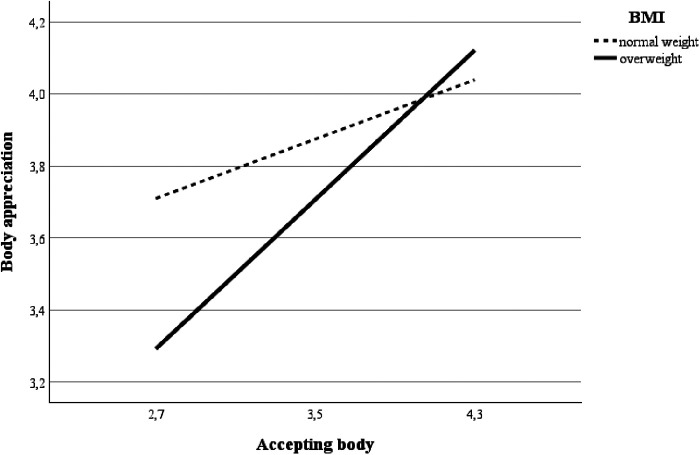
Moderating role of body mass index (BMI) on the association between accepting the body during the last exercise session and body appreciation.

No moderating roles of BMI were found between physical activity and both study mediators: accepting the body (*β *= 0.05, *p* = 0.696) and monitoring the body (*β *= –0.14, *p* = 0.291) during the exercise. Also, BMI did not moderate the direct association between monitoring the body during exercise and body appreciation (*β *= –0.37, *p* = 0.087). Importantly, BMI moderated the association (*β *= 0.61, *p* = 0.002) between accepting the body during the last exercise session and body appreciation (Figure 3). It was revealed that for overweight students, the association between accepting the body during the last exercise session and body appreciation was stronger (*B* = 0.53, *p* < 0.001) than for those with a normal BMI of <25.0 kg/m^2^ (*B* = 0.21, *p* < 0.001). Lastly, the direct association between physical activity and body appreciation was also moderated by the BMI (*β *= 0.26, *p* = 0.040): the association between physical activity and body appreciation was not significant for study subjects with normal BMI (*B* = 0.001, *p* = 0.351), but was significant for overweight (*B* = 0.01, *p* = 0.004).

**Figure 4:**
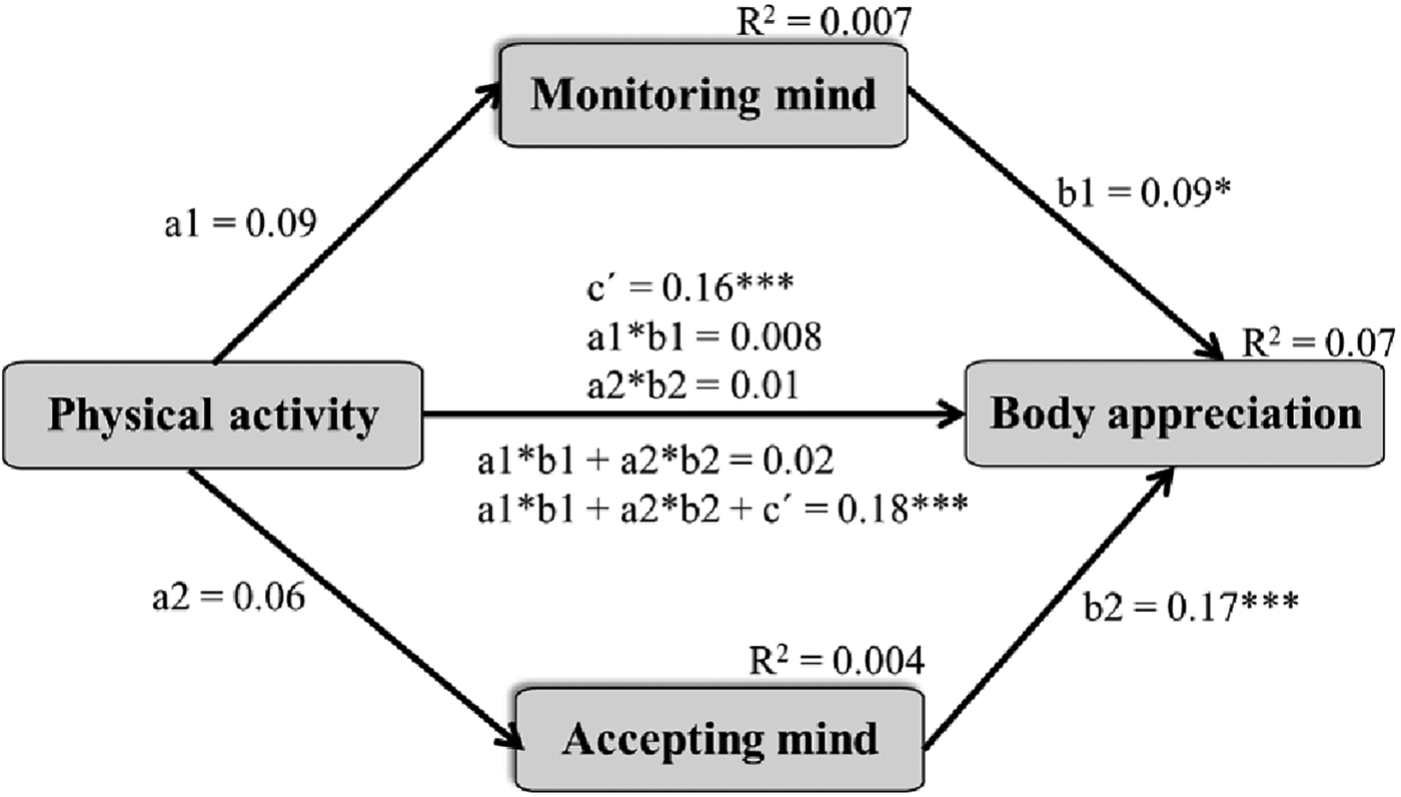
Mediated model of physical activity, mindfulness in monitoring and accepting the mind during exercise and body appreciation. * *p* < 0.05, **p < 0.01, *** *p* < 0.001 two-tailed; coefficients are standardised; a1—effect from the independent variable (**X**) on mediator 1 (M1), b1—effect from M1 on the dependent variable (**Y**), a2—effect from X on M2, b2—effect from M2 on Y, ć—direct effect from X to Y, a1*b1—specific indirect effect of X on Y via M1 only, a2*b2—specific indirect effect of X on Y via M2 only, a1*b1 + a2*b2—total indirect effect of X on Y via M1, M2, a1*b1 + a2*b2 + ć—total effect.

In the last step, a mediated model of PA, mindfulness in monitoring and accepting the mind during PA and body appreciation was tested. Due to weak correlations between the study variables, the calculated power for this model was inadequate (0.24–0.30) and the association between PA and accepting and monitoring the mind during exercise was not significant. The desired level of power is 0.80 or 80% probability of finding a significant result. Thus, accepting and monitoring the mind during the exercise did not mediate the path between PA and body appreciation (Figure 4). There was a direct association between PA and body appreciation and the associations between monitoring and accepting the mind during exercise and body appreciation were also significant. All information on direct, indirect and total effects is presented in Figure 4 footnotes. PA and monitoring and accepting the mind during exercise explained only 7% of the variance in body appreciation.

As the model demonstrated poor statistical power and no significant mediating roles of monitoring and accepting the mind during the last exercise in the association between physical activity and body appreciation, the moderating roles of sex and BMI were not additionally tested.

## 4 Discussion

The present study expands the knowledge of the possible mechanisms that explain the associations between PA, mindfulness and positive body image. Overall, the present results provided evidence that body-related mindfulness (monitoring and accepting the body) during PA is an important mediator between PA and body appreciation in physically active young adults of both sexes. Below we highlight how the results of the present study uniquely add to the literature assessing the relationships between PA, mindfulness and positive body image.

### 4.1 Mediating role of monitoring and accepting the body and mind in associations between physical activity and body appreciation

The results of the present study suggest and add important new knowledge indicating that participation in PA is associated with body appreciation not only directly but also through the two mediators of monitoring and accepting the body. This finding is in line with the Monitoring and Acceptance Theory (MAT), which postulated that attention monitoring skills enhance awareness of the present moment and affect positive cognitive outcomes, affective experience and reactivity and acceptance skills, all of which modify the way one relates to the present-moment experience, thus regulating reactivity to affective experience (30, 31). Specifically, our study adds important new knowledge that open and non-judgmental awareness (monitoring) and acceptance of physical body sensations during PA are associated with enhanced body appreciation. This new knowledge might inform PA and positive body image promotion-focused interventions, as well as prevention and treatment programmes for individuals with higher body image concerns (25, 50). Previous studies show that body image concerns and insecurity about the body are related to lower PA and the avoidance of PA (11, 12). Thus, increasing mindfulness in PA might enhance non-judgmental monitoring and acceptance of the body, which further increases a positive body image.

Previous studies showed that mindfulness-based physical activities such as yoga increase a positive body image (15, 17, 21, 22, 50). The present study adds important new knowledge that various physical activities might be beneficial to the body image of exercising young adults if body-related mindfulness (non-judgmental monitoring and acceptance of the body) is increased. It is important to acknowledge that the SMS-PA-2 did not let us assess the connections between body and mind, which is the central construct of embodiment (14, 16, 24). Based on the Embodiment Model of Positive Body Image (14, 16), embodying physical activities increases a positive body image through mediators such as embodiment (awareness and attentiveness to the body, a sense of physical empowerment, increased body and mind integration) and lower self-objectification (understanding and treating your body as an object) (14). Thus, increased embodiment or enhanced body and mind connection might be another important mediators through which PA, mindfulness and positive body image might be interrelated in young exercising adults (14, 24). In future studies we recommend testing mindfulness in PA using instruments that allow the connection between mind and body to be assessed (16).

In the present study, we observed no mediating role of monitoring and accepting the mind in the associations between PA and body appreciation. We also observed lower correlations between monitoring and accepting the mind and body appreciation than between monitoring and accepting the body and body appreciation. An explanation for these results might be that monitoring and accepting the mind in PA has lower or minimal significance for body appreciation. PA and sports are contexts in which the body has a central focus; and monitoring and accepting the mind are more distal variables to the body compared to monitoring and accepting the body in PA contexts. Furthermore, young and physically active adults participated in the present study. It might be that people who exercise feel less mental and/or psychological discomfort during PA because of their higher adaptation to PA and increased interoceptive awareness (afferent sensing, central processing and mental representation of the internal body signals) compared to the sedentary population (51). Therefore, it is important to investigate samples with lower PA or those not constantly exercising in future studies. However, because this study is one of the first to test the associations between general PA, mindfulness during exercise and body appreciation, our findings could be random. We therefore recommend that future studies test this model of PA and body appreciation on different samples.

### 4.2 Moderating role of sex and body weight in the associations between physical activity, mindful body acceptance and body appreciation

In the present study, we also tested the moderating role of sex and BMI in the associations of the model. No moderating role of sex were observed, suggesting that the associations between PA and mindfulness and monitoring and associations between acceptance of the body with body appreciation are similar in both sexes. The sample of the present study consisted of young physically active adults and physical activity, body appreciation as well as interoceptive awareness in women of our sample might be higher compared to women from the sedentary populations (11). It is possible that we did not observe differences for women and men because of the ceiling effect. Nevertheless, it is one of the first studies, and future research should be continued to have more knowledge on this topic.

However, our study showed that the associations between body acceptance during PA and body appreciation are stronger in overweight and obese participants than in participants of normal body weight. This is important new knowledge showing that mindfulness in PA, especially body acceptance, might be more beneficial for positive body image development in overweight and obese people compared to individuals with normal body weight. Overweight individuals report higher negative body image and/or body image concerns and less positive body image (34, 52, 53), therefore they more often experience negative body sensations during PA (i.e., body shame, fatigue, pain, exertion). These negative body-related feelings might prevent them from PA or even promote resistance to it (54). Thus, the results of the present study show that enhancing mindfulness in PA, especially acceptance of the body, might help individuals with higher BMI and/or body image concerns to develop a more positive body image and to sustain PA (4).

Importantly, no moderating role of body weight were observed in testing other associations of the hypothetical model. For example, BMI was not a moderator in the associations between monitoring of the body and body appreciation. This is an important finding suggesting that mindful acceptance, but not mindful monitoring, of the body is an important variable for the development of positive body image in individuals with higher body mass. These results inform intervention programmes for obesity prevention and treatment and might suggest that for individuals with higher body image concerns, mindful acceptance of the body should be emphasized during PA.

### 4.3 Practical implications

The findings of the present study might inform PA, positive body image promotion-focused interventions and prevention and treatment programmes for overweight individuals. Including mindfulness training in sports and PA practices, as well as teaching sport and health and fitness coaches to implement strategies enhancing mindfulness, might help exercisers to develop more healthy attitudes towards their body image. These findings also might inform obesity treatment interventions, suggesting that integration of sports practices that include mindful monitoring and acceptance of the body might help individuals with higher body weight to develop a positive body image as an outcome of physical exercise. Positive body image is related to more mindful eating and lower body weight (55–57), therefore these programmes might also help overweight individuals to decrease excessive body weight more effectively compared to general PA programmes in which mindfulness is ignored.

### 4.4 Limitations and strengths of the present study

The main limitation of the present study is its cross-sectional design, which did not allow us to understand the directions of the associations between the study variables. It might be that individuals with a more positive body image demonstrate higher mindful body monitoring and acceptance skills and therefore are more physically active. Another limitation is that we did not assess the duration of previous sports participation in the present study. A longer sports experience might be an important moderator that changes the strength of the associations between PA, mindfulness during exercise and positive body image. In other words, in longer exercising individuals the associations between monitoring and acceptance of the body and body appreciation might be higher compared to novice exercisers. We recommend testing this assumption in future studies.

Another limitation was that state mindfulness was not assessed immediately after the exercise session but some time later, asking participants to recall the last exercise session. Half of the exercisers reported that they exercised on that or the previous day; however, 14% of the sample recalled that the last workout was more than a week ago. This is an important limitation and in future studies we recommend that state mindfulness be assessed immediately after the exercise session, as recommended by the authors of the SMS-PA-2 (29). We also recommend assessing satisfaction with body, body functionality appreciation, enjoyment of PA and interoceptive awareness in future studies. These variables might be important for testing the associations between PA, mindfulness and positive body image (17, 51, 58–60).

The main strength of the present study was that we tested the associations between state mindfulness and PA. Previous systematic research reported a large gap in the literature on this topic (3). Another strength is that we assessed state mindfulness in a sample of young adults practicing various sports, including organized individual and team sports, recreational exercise and exercising at home. Previous studies tested the role of mindfulness for body image in small specific samples of exercisers (i.e., yoga). In contrast to studies assessing only one type of sport, testing the sample participating in various sports let us to see a general and complex picture of the mediating role of mindfulness in the associations between PA and positive body image. A further strength of the study was that we explored a sample of physically active individuals that included both sexes. Finally, an important strength of the present study is that we used internationally sound measures such as the BAS-2 and SMS-PA2.

## 5 Conclusions

The results of the present study show that mindful monitoring and acceptance of the body during PA mediate the associations between PA and positive body image (operating as body appreciation) in a sample of young physically active adults. Monitoring and accepting the mind did not mediate these associations. The associations between mindful acceptance of the body and positive body image were stronger in individuals with higher body mass compared to those with a normal body mass index. These findings might inform positive body image promotion programmes suggesting that using coaching methods that increase mindful monitoring and acceptance of the body during exercise might help to develop a healthier body image in exercisers and enhance more inclusive exercise environments in the sports and recreational exercise sectors.

## Data Availability

The original contributions presented in the study are included in the article/**Supplementary Material**, further inquiries can be directed to the corresponding author.
